# Fabrication of TiN Coatings Deposited on Laser Shock Micro-Textured Substrates for Improving the Interface Adhesion Properties of Coatings

**DOI:** 10.3390/ma17133302

**Published:** 2024-07-04

**Authors:** Ying Xu, Yixin Chen, Dongcheng Zhou, Lei Zhang, Boyong Su

**Affiliations:** 1 Engineering Training Centre, Nantong University, Nantong 226019, China; xuying@ntu.edu.cn (Y.X.); aimi614@ntu.edu.cn (Y.C.); 2 Zhejiang Dahua Technology Co., Ltd., Hangzhou 310000, China; 3 School of Mechanical Engineering, Nantong University, Nantong 226019, China

**Keywords:** laser shock peening, TiN coating, surface texturing, residual stress, interfacial adhesion, friction and wear

## Abstract

This paper aims to investigate the strengthening mechanism of laser shock peening on the interfacial bonding properties between TiN coatings and TC4 titanium alloy substrates. The different surface textures were induced by LSP on a TC4 titanium alloy substrate. Subsequently, titanium nitride (TiN) coatings were deposited on the surface texture. A scratch test and reciprocating sliding wear assessment were conducted to evaluate the impact of LSP on the interfacial bonding properties and wear performance of the coatings. The experimental results demonstrated that the adhesion of TiN coatings deposited on the surface texture formed by laser shock peening was significantly enhanced. The efficacy of laser shock treatment in reducing wear rates was found to be significantly enhanced in cases of both increased spot overlapping rate and increased laser power density. The surface texture created using laser parameters of 6.43 GW/cm^2^ and a 50% overlapping rate was found to have the most significant effect on improving the adhesion and anti-wear properties of the coating. The laser shock texture was identified as the main contributor to this improvement, providing a large interfacial contact area and a mechanical bond between the coating and the substrate. This bond inhibited the initiation and propagation of micro-cracks caused by the concentration of internal stress and interfacial stress of the coating.

## 1. Introduction

TC4 titanium alloy has been a popular choice in the fields of shipbuilding, petrochemicals, aviation, and energy due to its high specific strength, good fatigue strength, high fracture toughness, and corrosion resistance. However, TC4 titanium alloy exhibits relatively poor tribological properties such as low hardness (micro-hardness was ~18.6HRC_5_) and unstable coefficient of friction [1,2,3,4,5]. Consequently, surface coatings (TiN, TiAlN, etc.) deposited by physical vapor deposition (PVD) have been widely used to improve the performance of TC4 titanium alloy because of their excellent high-temperature oxidation resistance, corrosion resistance, and wear resistance [6,7,8].

Due to the mismatch between the mechanical properties of the PVD coatings and the substrate, the coatings often encountered the problem of insufficient adhesion strength, which makes them prone to cracks and peeling under actual conditions of use [9]. In order to address the aforementioned issues, a number of advanced techniques have been employed throughout the coating manufacturing process. These include the optimization of coating deposition parameters [10], the adjustment of coating chemical composition [11,12], and the pre-treatment of substrates [13,14]. 

In recent years, extensive research has been conducted on the effects of pre-treatment on PVD coatings [15], including shot peening, laser processing, ultrasonic rolling, and other techniques. The implementation of pre-treatment measures can result in the generation of a high degree of flexibility in the geometry, an increase in the contact area, and the provision of a suitable bonding interface. Nevertheless, the aforementioned pre-treatment techniques are not without shortcomings. For instance, shot peening is known to exhibit poor uniformity, laser processing can cause heat effects, and ultrasonic rolling can result in a shallow depth of the affected layer. [16]. Consequently, the manufacture of an efficient and high-quality surface texture was of paramount importance in order to enhance the performance of the coating interface. In light of these considerations, Fan et al. proposed a micro-texturing method based on the laser shock peening technique [17]. Sarvesh Kumar Mishra et al. employed laser shock strengthening treatment to purify the substrate prior to fabricating AlTiN and AlCrN coatings. The findings demonstrated that the combined application of LSP and coatings could diminish the extent of wear and enhance the wear resistance of the substrate [18]. These results demonstrated that LSP could not only induce residual compressive stress but also fabricate uniform micro-textures on the surface of the material. However, the effects of micro-textures on the multi-scale relationship of microstructure have not been fully explored. Consequently, it was imperative to examine the combined impact of laser shock texture (LST) pre-treatment and coating deposition on the interfacial adhesion properties of hard coating/mild steel substrates.

In this paper, laser shock texturing (LST) was employed to fabricate micro-textures on a TC4 titanium alloy surface, with the objective of enhancing the adhesion properties of TiN coatings. The surface characteristics, including surface residual stress, micro-hardness, and surface topography, were measured. The effects of TiN coatings with/without laser shock micro-textures on the bonding performance and wear behaviours were examined. The strengthening mechanism of LSP on the bonding interface was also illustrated.

## 2. Experimental Procedure

### 2.1. Fabrication of TiN Coatings Deposited on LSP Textured Substrates

A TC4 titanium alloy was employed as the substrate, with dimensions of 30 mm × 30 mm × 3 mm. Prior to the LSP pre-treatment, the specimen surfaces were polished with SiC paper of varying degrees of roughness (200 #, 400 #, 600 #, 1000 #, 2000 #, 3000 #). Subsequently, the specimen surfaces were cleaned by irradiating them with ultrasound in ethanol. The LSP pre-treatments were conducted using a Q-switched Nd:YAG laser operator (Procudo200; LSPT, Ltd., Houston, TX, USA). The repetition rate was set to 5 Hz, and the wavelength was taken to be 1064 nm. During the LSP process, water was used as the transparent confining layer, while black polyester tape was used as the absorbing layer. The thickness of the transparent confining layer and absorbing layer were approximately 1 mm and 180 µm, respectively. The LSP pre-treatment parameters are presented in Table 1.

Following the LSP micro-texturing, the treated samples were subjected to ultrasonic washing with acetone and absolute alcohol for a period of 10 min in order to remove any residual impurities. The TiN coatings were then deposited on the different treated substrate surfaces by means of an ion plating treatment (NANOARC-SP1010; Naarc New Material Technology Co., Ltd., Wuxi, China). During the coating fabrication process, titanium with a purity of 99.99% was selected as the target material. The sample was mounted on the turret of the vacuum chamber, with a turret frequency of 20 Hz. The vacuum chamber was evacuated to a pressure of approximately 4.0 Pa, before being further evacuated to 4.0 × 10^−3^ Pa. The heating temperature of the vacuum chamber was set to 80 °C. During the surface activation process, Ar gas was introduced at a flow rate of 400 standard cubic centimetres per minute (sccm), a pulse bias voltage of 600 volts (V), and a duty ratio of 50%. The surface of the TC4 titanium alloy sample was cleaned with Ar ion glow for 15 min.

In order to enhance the coating–base bonding force and the coating stress, a titanium (Ti) layer was deposited as a bottom layer. The pulse bias voltage was set to 150 V, the duty cycle was set to 15%, and the working pressure was 0.8 Pa. The arc current was 100 A, and the deposition time was 5 min. In the fabrication of TiN coatings, N_2_ gas with a purity of 99.99% was introduced into the vacuum chamber, with the N_2_ gas flow set to increase in a stepwise manner. The N_2_ gas flow was 0→10 sccm for 5 min and 10→30 sccm for 20 min. At the point at which the N_2_ gas flow reached 30 sccm, it was maintained at this level.

### 2.2. Coating Performance Test

Following the sample preparation, the surface topography was quantified using the Veeco Wyko NT 1100 non-contact optical profiler (NCOP) (NT1100; Veeco Inc., Tucson, AZ, USA). Surface roughness was characterized according to Equations (1)–(3) [19,20] as follows:

(1) $$Ra=\frac{1}{lr}\int_{0}^{lr}\left|Z(x)\right|dx$$

(2) $$R_{y}=\left|z_{\max}-z_{\min}\right|$$

(3) $$Rz=\frac{1}{5}\left[\left|\sum_{i=1}^{5}{\left(z_{i}\right)}_{\max}+\sum_{j=1}^{5}{\left(z_{j}\right)}_{\min}\right|\right]$$

where Ra is average roughness, $R_{y}$ is peak-to-valley height roughness, Rz is 10-point roughness, *lr* is assessment length, *Z*(*x*) is surface height for Z = 0, and $z_{\max}$ and $z_{\min}$ are the five higher local maxima and lower local minima of the profile height distribution (z), respectively. 

The residual stress was determined using the X-ray residual stress tester (Pro-to-LXRD; Proto Manufacturing Ltd., Windsor, ON, Canada). The X-ray beam diameter was approximately 1 mm. The X-ray source was a Cr/Kα ray, and the diffraction plane was a phase (310) plane in the stress calculation. The feed angle of the ladder scanning was 0.1°/s. The scanning starting angle and terminating angle were 137° and 144°, respectively. The measurement area is situated in the centre of the single spot. Each measurement was repeated five times for each condition, and after each measurement, the tested sample was rotated around the centre of the tested point. The average value was used.

The micro-hardness was quantified using a micro-hardness tester (MKV E3, Mitutoyo, Kawasaki, Japan) under an applied load of 1 N (100 gf) for a constant indenter dwell time of 15 s. To ensure the reproducibility of the data, each sample was measured at least five times to obtain the mean value.

The scratch method was employed to assess the interface bonding performance of the TiN coating and TC4 titanium alloy. The instrument utilized was a WS-2005 film adhesion automatic scratch tester (Zhongke Kaihua Technology Development Co., Ltd., Lanzhou, China). During the test process, the dynamic load perpendicular to the surface was 50 N, the loading rate was 50 N/min, and the scratch length was 4 mm. The measurement method employed a single reciprocating approach, whereby acoustic emission signals were received and subsequently combined with friction change and scratch morphology data. This enabled the critical load value of the coating to be determined. Each sample was measured on three occasions, with the average value subsequently calculated.

The wear resistance of the sample was evaluated using the TBT-M5000 friction and wear tester (Rtec Instruments Inc., San Jose, CA, USA) at a temperature of approximately 20 °C, a humidity of approximately 50%, and an atmospheric pressure of 1 atm under dry friction conditions. An Si_3_N_4_ ceramic ball with a diameter of 10 mm was selected as the upper counterpart. The ball-disc reciprocating test was selected with a normal load of 50 N, a reciprocating stroke of 5 mm, and a reciprocating frequency of 2 Hz. Following the wear performance test, the specimen surfaces were cleaned by irradiating with ultrasound in ethanol. A scanning electron microscope (SEM) (Hitachi S-3400N, Hitachi High-Tech Co., Ltd., Shanghai, China) was employed to observe and detect the different samples, and subsequently analyze the wear mechanism.

## 3. Experimental Results and Analysis

### 3.1. The Results of LSP Pretreatment

**Figure 1:**
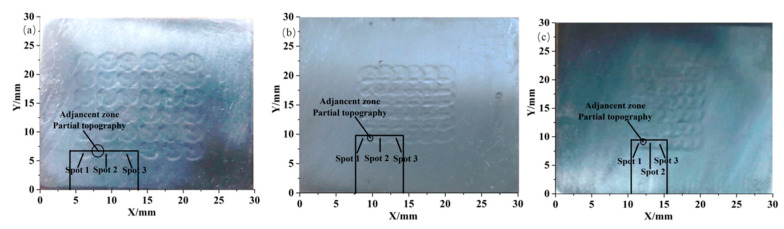
The distribution of micro-pits on the substrate after treatment with three different overlapping rates: (**a**) 0; (**b**) 33%; (**c**) 50%.

Figure 1 presents the surface morphology of the peened surface after LSP under different overlapping rates (laser power density: 3.02 GW/cm^2^; pulse width: 20 ns; spot diameter: 2.5 mm.) The overlap ratios in Figure 1a–c were 0, 33%, and 50%, respectively.

As illustrated in Figure 1, the surface morphology of the substrate underwent a significant alteration following the LSP treatment. The formation of a micro-indentation with a certain depth resulted in a convex deformation on the edge of the micro-indentation. This phenomenon can be attributed to the absence of additive and reduction of material during LSP processing. Instead, the material was subjected to a process of squeezing towards the adjacent areas. The formation of the micro-indentions and convex deformation were induced by the combined effects of continuous extrusion, stretch, compression, shear, and impact. Furthermore, it was noteworthy that the micro-indentions and convex deformation were evenly distributed and arranged on the treated surfaces, which confirmed the controllability of LSP texturing. 

**Figure 2:**
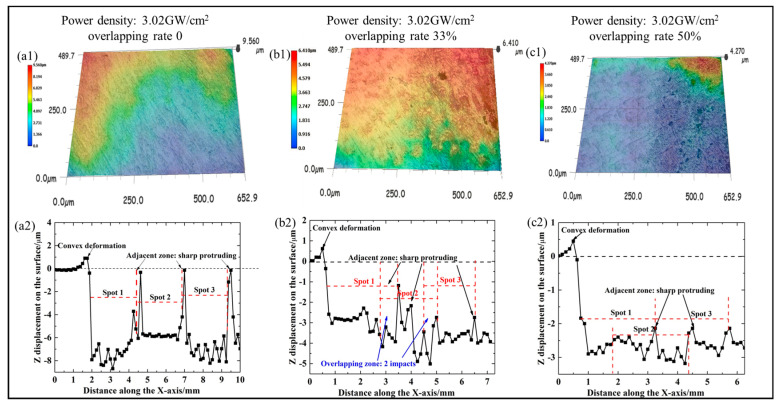
(**a1**,**b1,c1**) Surface morphology; (**a2**,**b2**,**c2**) plastic deformation distribution at different overlapping rates on the substrate.

Figure 2 displays the three-dimensional surface texture of samples at 3.02 GW/cm^2^ and different overlapping rates. As illustrated in Figure 2(a1), the laser shock peening treatment resulted in a change in the geometrical topography of the sample when the overlapping rate was 0. This was evident in the micro-pits observed in the centre of the spot, as well as the bumps at the junction of the spot and the spot. The height difference was approximately 9.6 μm. Figure 2(a2) illustrates the height change along the X-axis at 3.02 GW/cm^2^ and 0 overlapping rate. It can be observed that the laser shock treatment resulted in the formation of an indention with a depth of approximately 8 μm in the centre of the spot and a bump with a height of 1.6 μm at the junction of the spot. The change in surface roughness was directly correlated with the micro-indentation and convex deformation. According to Equations (1)–(3), the average deviation Ra of the sample surface profile was 4.99 μm, the microscopic unevenness Rz was 3.92 μm, and the maximum profile height Ry was 9.6 μm.

The results of the surface geometry analysis for the overlapping rate of 33% are presented in Figure 2(b1,b2). Figure 2(b1) illustrates the height difference of the overlapping area, which was found to be 6.5 μm. Figure 2(b2) depicts the different areas subjected to different times of laser shock. When the overlap rate was 33%, the height of the protrusion deformation was significantly reduced. However, due to the different number of impacts received in different areas, the amplitude of surface deformation increased significantly. According to Equations (1)–(3), the average roughness Ra was 2.99 μm, the peak-to-valley height roughness Ry was 6.2 μm, and the 10-point roughness Rz was 2.23 μm, respectively.

The surface geometry results of an overlapping rate of 50% are demonstrated in Figure 2(c1,c2). At this juncture, the area designated as spot 2 was overlapped by spots 1 and 3. The overlapping zone was subjected to two and three impacts, respectively. Upon completion of the three treatments, the treatment effect was deemed to have reached its saturation point [21]. The detailed distribution of the surface deformation demonstrated that the formation of sharp protrusions was exclusive to the non-overlapping zone, with the height of these protrusions decreasing to 2.752 μm. The surface roughness exhibited good uniformity in the overlapping zone. According to the Equations (1)–(3), the average roughness (Ra) was 2.29 μm, the peak-to-valley height roughness (Ry) was 3.63 μm, and the 10-point roughness (Rz) was 1.46 μm.

**Figure 3:**
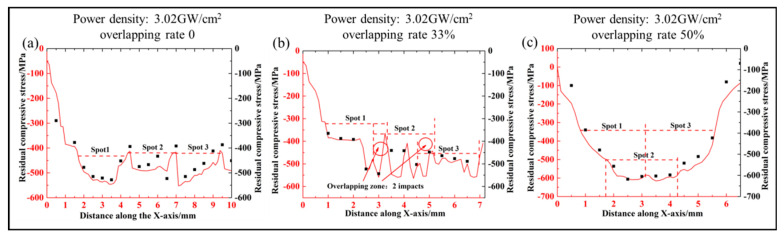
Surface residual stress distribution at different overlapping rates on the substrate, (**a**) overlapping rate 0; (**b**) overlapping rate 33%; (**c**) overlapping rate 50%.

Figure 3 presents the surface residual stress distribution at varying overlapping rates. Figure 3a illustrates the distribution of residual stress on the sample surface when the overlapping rate was 0. The residual stress results also demonstrated that the residual stress distribution was uneven, which was consistent with the surface morphology distribution. The residual compressive stress on the surface of the material exhibited a distribution pattern of “low in the middle and high at both ends”. This indicated that a larger residual compressive stress was formed in the centre of the spot with the LSP treatment, gradually decreasing towards the edge. The mean residual compressive stress following treatment with an overlapping ratio of 0 was −435 MPa. The results demonstrated that the surface geometry and residual compressive stress of the sample were directly influenced by the plastic deformation of the material surface and the transfer of the compressed volume following laser shock treatment. Figure 3b illustrates the distribution of residual compressive stress on the sample surface when the overlap ratio was 33%. Figure 3b illustrates that the residual compressive stress in the overlapping area was approximately −500 MPa, while the residual compressive stress in the non-overlapping area was approximately −400 MPa. This indicated that increasing the number of impacts can effectively increase the surface residual stress. The mean residual compressive stress following multi-point treatment with an overlap ratio of 33% was −472 MPa. Figure 3c illustrates the distribution of residual compressive stress in the local area when the overlapping rate was 50%. As illustrated in Figure 3c, the residual compressive stress in the overlapping area was approximately −600 MPa, while the residual compressive stress in the non-overlapping area was approximately −460 MPa. The mean residual compressive stress following multi-point treatment with a 50% spot overlap rate was −539 MPa.

**Figure 4:**
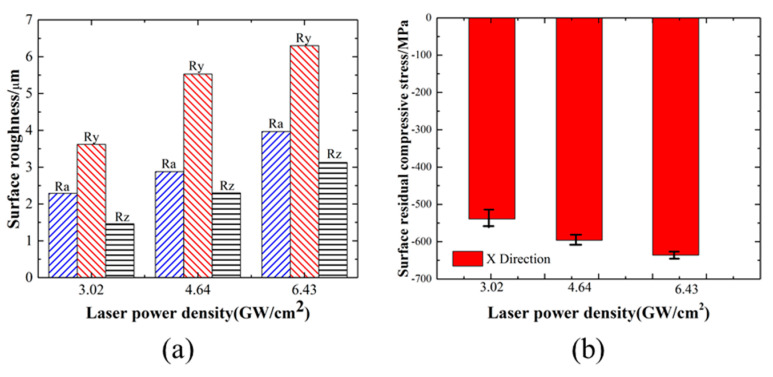
(**a**) Surface roughness; (**b**) residual stress on the substrate with different power densities.

Figure 4 presents the results of surface roughness and residual compressive stress at different power densities. From the aforementioned analysis, it was evident that the surface roughness and residual stress distribution of the sample exhibited a relatively uniform pattern when the spot overlapping rate was 50%. At this juncture, alterations to the laser power density will directly impact the shock wave intensity, thereby resulting in the formation of disparate geometric morphologies and residual stress distributions on the surface of the material. The samples were subjected to treatment with laser powers of 3.02 GW/cm^2^, 4.64 GW/cm^2^, and 6.43 GW/cm^2^ (spot diameter of 2.5 mm, overlap rate of 50%, pulse width of 20 ns).

As illustrated in Figure 4, at a laser power density of 3.02 GW/cm^2^, the average roughness Ra was 2.29 μm, the peak-to-valley height roughness Ry was 3.63 μm, the 10-point roughness Rz was 1.46 μm, and the average residual stress of the surface was −539 MPa. When the laser power density was 4.64 GW/cm^2^, the average roughness Ra was 2.88 μm, the peak-to-valley height roughness Ry was 5.53 μm, the 10-point roughness Rz was 2.3 μm, and the average residual stress of the surface was −596 MPa. When the laser power density was 6.43 GW/cm^2^, the average roughness Ra was 3.97 μm, the peak-to-valley height roughness Ry was 6.3 μm, the 10-point roughness Rz was 3.13 μm, and the average residual stress was −636 MPa, respectively. The results demonstrated that as the laser power density increased, the surface roughness and residual compressive stress of the material following laser shock strengthening treatment exhibited a gradual increase.

Table 2 presents the surface hardness following treatment with varying laser parameters. As illustrated in Table 2, laser shock strengthening treatment also exerted a beneficial influence on surface hardness, exhibiting a comparable effect to that observed for residual stress on the material surface. Furthermore, as the spot overlapping rate and power density increased, surface hardness exhibited a gradual enhancement. The optimal treatment effect was observed when the spot overlapping rate was 50% and the power density was 6.43 GW/cm^2^. This resulted in a surface hardness of 253 HV0.1. The positive effect of laser shock peening treatment on the hardness of the material can be attributed to the ultra-high strain rate plastic deformation of the surface structure under the action of the laser shock wave, which resulted in the formation of more dislocations and the refinement of the grains [22,23].

### 3.2. Coating Interface Bonding Performance

**Figure 5:**
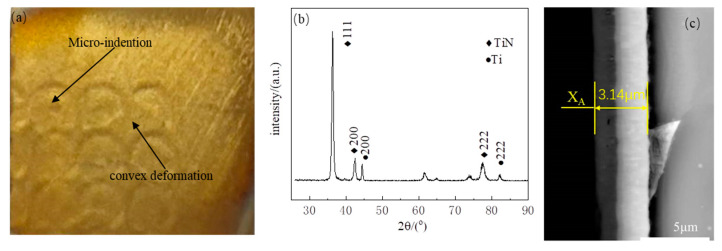
(**a**) The TiN coatings deposited on the substrate; (**b**) XRD pattern of TiN coatings; (**c**) the cross-section morphology of TiN.

Figure 5 presents the TiN coatings deposited on the A1 sample. Figure 5a illustrates that the micro-indentation and convex deformation remained on the substrate, indicating that the ion plating technology did not regulate the surface profile of the substrate. Figure 5b displays the XRD pattern of TiN deposited on the substrate. During the acquisition process, a small incident angle of 0.6° was employed to reduce the X-ray incidence depth, thereby reducing the influence of the substrate on the analysis of the coating. As illustrated in Figure 5b, the TiN coating comprises two phases, namely Ti and TiN. TiN exhibited a strong (111) preferred orientation structure. Figure 5c presents the cross-sectional morphology of TiN, as observed via scanning electron microscopy (SEM). The coating thickness was estimated to be approximately 3.14 μm. Table 3 displays the coating thickness of A2~A5, demonstrating that the thickness differed by less than 1%, indicating that laser shock textures had no significant effect on the coating thickness.

**Figure 6:**
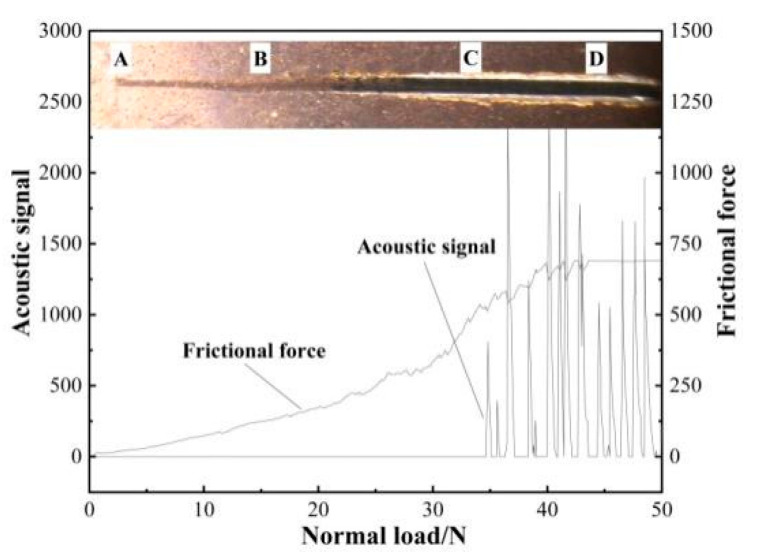
Acoustic and friction signals, optical micro-topography of TiN/substrate scratches.

Figure 6 illustrates the acoustic signal, friction signal, and optical microscopic morphology of the scratch obtained from the TiN coating scratch experiment. Currently, the most commonly employed techniques for evaluating the adhesion of the film base include the indentation method, the stretching method, and the scratch method. Among these methods, the scratch method was the most commonly used and provided a more effective means of testing the adhesion of the hard film layer [24]. The WS-2005 coating adhesion automatic scratch tester was employed in the experiment. When the 120° cone-shaped diamond indenter on the scratch tester was scratched on the coating with a continuous normal load, the membrane ruptured as the normal load increased to a certain value, accompanied by the generation of a sudden acoustic signal and friction force. At this juncture, the pertinent normal load is the coating-substrate bonding force.

As illustrated in Figure 6, in the AB section, due to the overall low normal load, the acoustic signal detected by the scratcher exhibited no discernible abrupt change. As the load was increased from a low to a high value, the cone angle indenter made a gradual penetration from the surface to the interior of the coating. The width of the scratches exhibited a transition from narrow to wider, accompanied by a corresponding increase in the friction detected by the test system. At this juncture, the coating remained intact. In the BC section, as the normal load increased, the width of the scratches increased, and a slight peeling of coating debris was induced at the edges of the scratches. At the point where the scratch reached C, the width of the scratch remained constant, yet the friction rose sharply as the indenter deepened, accompanied by a sudden change in the acoustic signal. Furthermore, the film exhibited a tendency to avalanche on both sides of the scratch. At this juncture, the corresponding normal load is 38.9 N, which equates to the measured coating–base bonding force LC = 28.9 N. As the load continued to increase, the scratch reached the CD segment, which was situated at the two-thirds point of the length of the starting point of the scratch. The abrupt change in the acoustic signal became more pronounced, and the frictional force reached the upper limit of the instrument’s capacity. These observations indicated that the cone angle indenter of the scratcher had made contact with the TC4 titanium alloy substrate. The coating–base adhesion force of the various samples is presented in Table 3.

As illustrated in Table 3, it can be demonstrated that the interface bonding force of the TiN coatings on the TC4 titanium alloy, which was pre-treated by LSP, was effectively improved in comparison to the untreated sample. The relative increase rates of the A1~A5 samples were 11.76%, 14.88%, 20.76%, 27.34%, and 37.03%, respectively. It can be observed that the interface bonding force increases with an increase in the overlapping rate and laser power density.

### 3.3. Friction and Wear Performance

**Figure 7:**
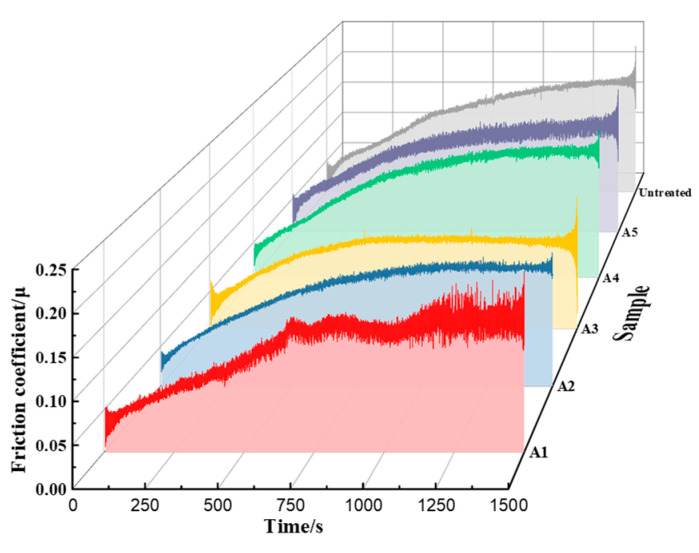
Friction coefficient graph of TiN/substrate.

The impact of LSP pre-treatment on the anti-adhesive wear performance of the TiN coatings was evaluated under friction conditions. Figure 7 illustrates the variation in the friction coefficient for Si_4_N_3_ balls reciprocating against the samples. It can be observed from the figure that the slope of the curve during the running-in period was significant, and the friction coefficient increased rapidly. In a relatively short period of time, a larger amount of wear was generated on the surface of the sample, which was mainly due to the convex deformation on the surface. The convex deformation resulted in a small contact area of the friction pair and strong local pressure.

Furthermore, Figure 7 indicates that the length of the running-in period for the six groups of samples in this experiment was approximately the same. In the subsequent phase, the wear rate of the specimen decreased significantly, while the friction coefficient remained relatively stable, indicating that the wear process had entered a stable stage. 

**Figure 8:**
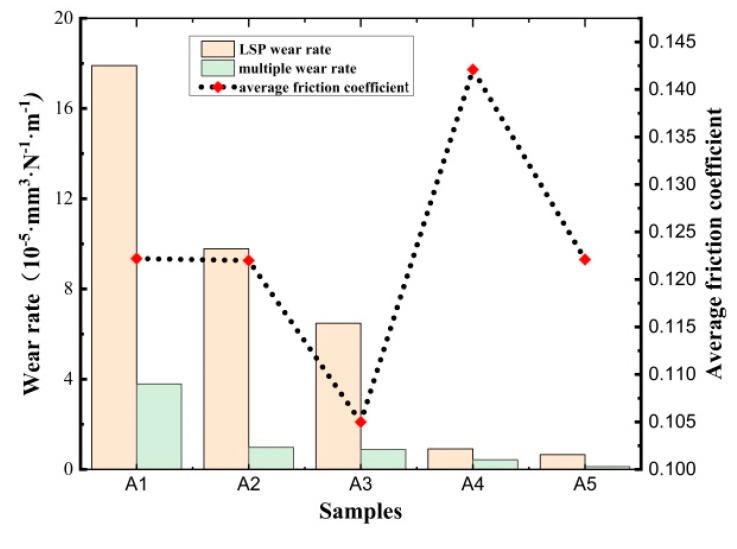
Average friction coefficient and wear rate of TiN/substrate.

The calculated wear rate, derived from mass loss and the coefficient of friction, is presented in Figure 8. The friction coefficient of the untreated TC4 titanium alloy substrate was 0.1612, and the wear rate was 3.32 × 10^−3^ mm^3^·N^−1^·m^−1^. As illustrated in Figure 8, it can be observed that the friction coefficient and the wear rate of the A1~A5 samples exhibited a reduction. The reduction rate of the wear rate was 94.6%, 97.1%, 98.04%, and 99.7%, 99.8%, respectively. The results indicated that the effect of laser shock treatment on wear rate reduction increased with increasing overlapping rate and laser power density. Furthermore, it can be observed that the wear rate of the TiN coating deposited on the LSP-treated samples was at least two orders of magnitude lower than that of the untreated sample. The sample with LSP parameters (laser power density: 6.43 GW/cm^2^, pulse width: 20 ns, spot diameter: 2.5 mm, overlapping rate: 50%) exhibited the lowest wear rate (1.24 × 10^−6^ mm^3^·N^−1^·m^−1^), which was only three ten thousandths of the untreated sample. The sample with the laser power density (3.02 GW/cm^2^) and pulse width (20 ns), along with a spot diameter of 2.5 mm and an overlapping rate of 0, exhibited the highest wear rate (3.79 × 10^−5^ mm^3^·N^−1^·m^−1^), which was one percent of the untreated sample.

**Figure 9:**
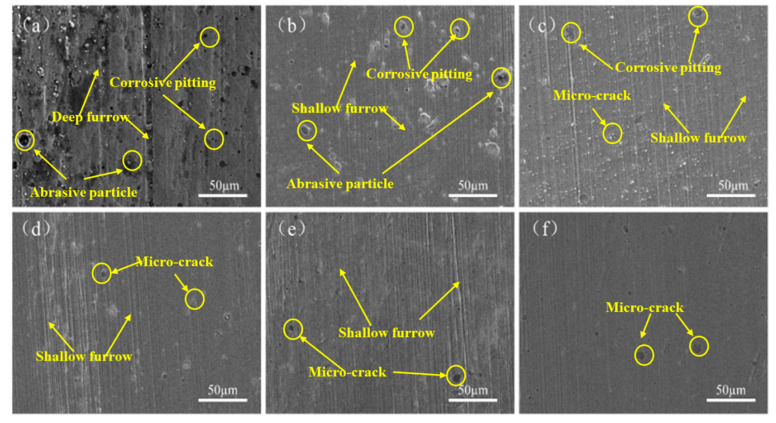
Wear topography of TiN/substrate: (**a**) untreated; (**b**) A1; (**c**) A2; (**d**) A3; (**e**) A4; (**f**) A5.

The SEM micrographs of the sample after a 1500 s sliding time are presented in Figure 9. It can be observed that the sample which did not undergo LSP pre-treatment exhibited extensive and deep spalling, micro-holes, and furrows. This may be attributed to the presence of micro-protrusions on the surface. Concurrently, the brittleness of the micro-protrusions was enhanced by the process of continuous loading and unloading, due to cold work hardening [25,26], which may result in brittle fracture of the micro-protrusions during the friction and wear test. Furthermore, during the brittle fracture process, abrasive particles were generated and pressed into the surface of the material. Furthermore, the abrasive particles also slid along the surface of the sample under the action of tangential force, causing micro-cutting and wear on the surface of the coating. The presence of furrow-like scratches on the surface of the coating was observed, as illustrated in Figure 9a. The presence of hard abrasive grains and wear marks on the coating surface resulted in the concentration of stress under the influence of cyclic loading, which in turn led to the generation and rapid expansion of micro-cracks. The micro-cracks may result in fatigue damage to the coating surface, which in turn may lead to the generation of a large number of pits and spalling. Furthermore, the dissimilar physical and mechanical properties of the TiN coating and the TC4 titanium alloy substrate, including the elastic-plasticity of the TC4 titanium alloy matrix, resulted in the generation of stress concentration at the interface and micro-cracks due to asynchronous deformation when subjected to the tangential force of the upper sample.

The wear status of samples A1–A5 is presented in Figure 9b–f. The wear morphology of the A1 sample, as illustrated in Figure 9b, demonstrated that the scratch was shallow and the wear condition was improved in comparison to that of the untreated sample. Nevertheless, a small area of peeling remained. Figure 9c depicts the wear morphology of sample A2. It can be observed that the surface wear was relatively minor, with the appearance of shallow furrow-like wear marks. It was observed that the coating surface exhibited evidence of abrasive wear. The wear morphology of samples A3 to A5, as illustrated in Figure 9d–f, exhibited a similar pattern. The worn surface exhibited a continuous and smooth appearance, with a notable reduction in the number of peeling and micro-pits, and the presence of only a few micro-cracks on the surface.

In general, the wear degree of samples A1–A5 was significantly less than that of the untreated samples, and the wear condition of A3–A5 was also superior to that of A1–A2. This was due to the fact that the micro-pits and micro-protrusions on the surface of the titanium alloy were evenly distributed as a result of the impact of a 50% spot overlap rate. The preceding analysis indicated that as the overlapping rate of samples A1–A3 increased, the surface effect became increasingly uniform following treatment. At this juncture, the primary factors influencing the interface bonding force were the micro-indentions and convex deformation induced by the LSP treatment. The improvement in the bonding force of samples A4–A5 was significantly greater than that of A1–A3. This phenomenon can be attributed to the fact that the material hardness and residual compressive stress were greatly improved compared with the previous groups of samples. However, the uniformity of the treatment effect was not destroyed under the condition of 50% overlapping rate. Following the deposition of the coating, the surface of the sample retained its regular and orderly texture. During the process of friction and wear, the anti-friction and lubrication effects were superior to those observed in samples A1 and A2, where the texture distribution of surface pits and protrusions was uneven.

### 3.4. The Strengthen Mechanism of LSP on the Bonding Interface 

Based on the comparison results of the physical and mechanical properties for the TiN coatings deposited on different treated surfaces, the surface texture implemented with laser parameters of 6.43 GW/cm^2^ and 50% overlap rate has the most significant effect on improving the adhesion and wear resistance of the coating. The probable enhancement mechanism of LSP texturing on the adhesion strength of the coating/steel substrate system is discussed below.

According to the interfacial surface morphology, the adhesion between the PVD coating and the substrate can involve three mechanisms, including mechanical bonding, physical bonding, and chemical bonding. Mechanical bonding was mainly influenced by the surface morphology. After the LSP process, the surface morphology of the material was reconstructed by the laser shock peening pre-treatment. Micro-indents and convex deformations were formed on the substrate surface during the LSP process, which increased the surface roughness. Provided that these surface textures were uniformly distributed, the bonding area between the substrate and the coating increased as the roughness increased. Thus, during the coating formation process, a mechanical bond was formed with these surface textures when the gas phase atoms entered the substrate surface [27,28], which improved the bonding performance of the coating and the substrate.

On the other hand, the influence of the internal stress of the coating and the residual stress at the interface junction on the interface bonding performance cannot be ignored. During the coating deposition process, due to the difference in thermal expansion coefficients between the coating and the titanium alloy substrate, the coating and the substrate have different thermal shrinkage tendencies, resulting in the corresponding stress at the coating interface junction [27,29,30]. Shear stress at the interfaces, micro-cracking, and coating failure were produced due to the internal stress. During the LSP treatment, the surface of the titanium alloy was deformed in the depth direction by the laser shock wave. The material was squeezed on both sides in a direction parallel to the surface. After the shock wave disappeared, the deformed layer still maintained a partially plastic deformation state, so the hardened layer and the residual compressive stress layer were induced on the substrate surface, which could improve the ability to resist elastic and plastic deformation. Then, during the coating growth process, the internal stress of the TiN coating and the stress concentration at the interface were eliminated [31], preventing the initiation and propagation of micro-cracks.

**Figure 10:**
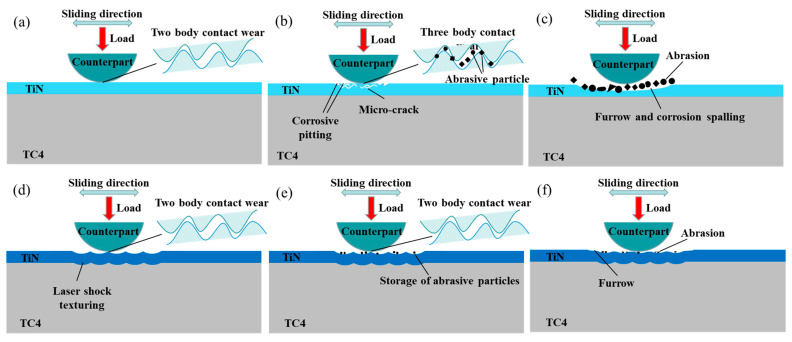
Schematic illustration of wear mechanism: (**a**–**c**) without LSP; (**d**–**f**) with LSP.

The improvement in the friction and wear performance of the TiN coating could be attributed to the improvement in the interfacial bonding performance between the coating and the substrate. Figure 10 shows a schematic illustration of the wear mechanism with and without LSP pre-treatment, in which the coating thickness and the substrate thickness were drawn to scale. As shown in Figure 1, the texture (micro-indentation and convex deformation) on the substrate induced by LSP increased the surface roughness (Figure 2), then the uneven micro-indentation and convex deformation increased the interface contact area between the coating and the substrate and promoted the micro-scale interaction on both sides of the interface, thereby forming a mechanical interlocking effect (Figure 10d). In addition, the non-uniform micro-deformation generated by the LSP could store wear debris and abrasive particles during the friction and wear process (Figure 10e), which could reduce the micro-cutting effect of foreign matter on the coating, thereby alleviating the abrasive wear on the coating surface (Figure 10f). However, for the samples without the LSP pre-treatment, the sample surface showed a severe wear morphology with a lot of adhesion spread on the worn surface (Figure 9a). The wear mechanism for the sample was considered to be the adhesion of the steel material to the TiN coating due to the low hardness of the grinding ball. During the wear process, the TiN coating had a large spalling area due to insufficient adhesion between the TiN coating and the substrate. Unlike the laser shock pre-treated specimens, the untreated specimens have no surface texture to hold randomly generated abrasive particles; the abrasive particles would aggregate into wear particles (Figure 10b,c).

In addition, for the samples with different LSP pre-treatment parameters, it can be observed from the test results that the contact area of sample A2~A3 decreased compared to that of sample A1, but the interfacial adhesion strength of sample A2~A3 continued to increase. Therefore, it can be concluded that the interfacial contact area does not seem to be the only factor that enhances the interfacial adhesion. Apart from the mechanical bonding caused by the surface topography, the improvement in bond strength could be attributed to the improvements in residual compressive stress and hardness, as confirmed by previous research [32,33]. Not surprisingly, the treated specimen also exhibited the highest surface residual compressive stress, which was in good agreement with the increase in hardness. The residual surface compressive stress could counterbalance the tensile stress generated during the coating deposition process. The increase in hardness could improve the ability to resist plastic deformation and provide more effective support for the TiN. When the coating was subjected to an external load, the mutual cancellation of the residual tensile and compressive stresses on the surface could effectively prevent the initiation and propagation of micro-cracks.

## 4. Conclusions

LSP was used to create micro-textures on the surface of the TC4 titanium alloy, then TiN coatings were deposited on the treated areas. After the LSP pre-treatment, surface properties including residual surface stress, micro-hardness, and surface topography were measured. The effects of TiN coatings with/without laser shock micro-textures on bonding performance and wear behaviour were investigated. Some conclusions are as follows:

(1) LSP could not only produce regular micro-textures on the surface of the titanium alloy, but also repeatedly regulate the surface quality. The residual compressive stress was induced in the micro-textured areas, the interfacial contact area was improved, and the internal grain structure was refined. The optimization of the above properties improved the interfacial bonding performance of the TiN coating.
(2) Different laser parameters, such as power density and overlap rate, mainly affected the distribution of surface micro-texture. The fabrication of TiN coatings deposited on a textured substrate treated with laser shock parameters of 6.43 GW/cm^2^ and a 50% overlapping rate results in the best interface bonding properties and anti-adhesive wear performance.
(3) The improvement in hardness could improve the ability to resist plastic deformation and give TiN more effective support. When the coating was subjected to an external load, the mutual cancellation of the residual tensile and compressive stresses on the surface could effectively prevent the initiation and propagation of micro-cracks.
(4) The surface texture formed by laser shock played a role in storing wear debris during the friction process and inhibited abrasive wear. The synergistic effect of laser shock and TiN coating improved the friction and wear performance of the material.

## Figures and Tables

**Table 1:** The detailed parameters of laser shock pre-treatment.

Laser Parameter	A1	A2	A3	A4	A5
Laser power density (GW/cm^2^)	3.02	3.02	3.02	4.64	6.43
Pulse duration (ns)	20	20	20	20	20
Spot diameter (mm)	2.5	2.5	2.5	2.5	2.5
Impact time	1	1	1	1	1
Overlapping rate (%)	0	33	50	50	50

**Table 2:** Surface hardness after treatment with different parameters.

	A1	A2	A3	A4	A5
Hardness (HV)	163	171	188	248	253

**Table 3:** Coating thickness and adhesion after treatment with different parameters.

	A1	A2	A3	A4	A5	Untreated
Thickness (μm)	3.14	3.18	3.12	3.16	3.11	3.10
Adhesion (N)	32.3	33.2	34.9	36.8	39.6	29.6

## Data Availability

Data are contained within the article.
